# Transparent Development of WHO Rapid Advice Guidelines: A Useful Approach

**DOI:** 10.1371/journal.pmed.0040245

**Published:** 2007-07-31

**Authors:** Antonio Cunha

I would like to congratulate Schünemann et al. for this very interesting and useful article [1]. In fact, proposing a systematic and transparent approach to developing guidelines may be useful not only for health problems that require rapid advice, but for any health problem, not necessarily emerging, including the existing ones in a certain setting. For instance, the proposed approach may be very useful for assessing health technologies, new ones or older ones not yet assessed, in many low-income countries.

Taking Brazil as an example, teams could be created in state- and/or municipality-level health secretariats to use this approach whenever a decision is needed regarding the purchase of drugs, products, or technologies or their inclusion in clinical guidelines. Local universities could collaborate in this process by suggesting names of expert faculties to join the panels, which would also increase credibility. In addition, health economists could support the process by advising on cost-effectiveness issues for the processes decided on. For this strategy to succeed, health authorities should coordinate the process and other stakeholders should be involved, such as, for instance, representatives of civil society patient associations.

In some specifi c circumstances, like the one presented in the article (uncertainty about the pharmacological management of avian infl uenza A H5N1 virus infection), the time frame to conclude the process is crucial. This might not be the case for several other circumstances, such as for example the decision to purchase aspirin or paracetamol to distribute in health centers freely (which occurs in Brazil) to treat children's common diseases with fever. The possibility of using this approach without time constraints makes it even more useful. An evaluation of the guidelines developed will still be necessary, to ensure their usefulness, and this could be conducted by the same team, this time coordinated by the university faculties already participating in the process.
